# Exploring the cost-effectiveness and optimization strategies of nursing caring for children and older adults: a perspective

**DOI:** 10.3389/fpubh.2026.1769664

**Published:** 2026-03-31

**Authors:** Wei Du, Chun-mei Gao, Yong Li, Ping Fu

**Affiliations:** 1 Department of Paediatrics, The Second Affiliated Hospital of Mudanjiang Medical University, Mudanjiang, China; 2 Department of Rehabilitation Medicine, The Second Affiliated Hospital of Mudanjiang Medical University, Mudanjiang, China; 3 Department of Medical Records, Hongqi Hospital Affiliated to Mudanjiang Medical University, Mudanjiang, China; 4 Department of Geriatric Medicine, The Second Affiliated Hospital of Mudanjiang Medical University, Mudanjiang, China

**Keywords:** child health, cost-effectiveness analysis, older adults care, health economics, integrated care model, nursing care, policy optimization, resource allocation

## Abstract

Children and older adults are two core groups in the nursing system with distinct needs and high economic dependence. The dual pressures of global aging and persistent childhood health issues strain nursing resources and raise concerns about long-term financial sustainability. Cost-effectiveness analysis provides a key framework for evaluating the economic efficiency of nursing interventions for these populations. It shows that child nursing acts as a forward-looking investment with significant long-term health and social returns, while care for older adults focuses on sustained management to maintain quality of life and alleviate caregiver burdens. To improve the nursing system, this perspective study proposes strategies based on integrated life-course care, precise resource allocation, technological support, and reforms in policy and financing. Implementing these approaches can enhance the efficiency of resource use and advance the equity, effectiveness, and sustainability of nursing care.

## 1 Introduction

Nursing care is a fundamental component of healthcare systems and is central to achieving universal health coverage (1). Globally, two major demographic shifts are converging: rapid population aging is increasing demands for chronic disease management and long-term support, while children face a growing burden of chronic conditions and developmental disorders requiring sustained care (2, 3). This dual challenge places significant pressure on limited nursing resources, making their allocation between these dependent populations a critical socioeconomic and policy issue (4, 5). The core dilemma is how to deliver care that produces meaningful health outcomes while ensuring long-term financial sustainability.

Analyzing the cost-effectiveness of nursing care for these two groups provides a vital pathway for improving resource allocation (6, 7). Although both require long-term care, their economic profiles differ substantially (6, 8). Care for older adults often focuses on late-stage medical interventions, functional support, and palliative care, with benefits primarily related to quality of life at the end of life (9). In contrast, investments in children-especially in prevention, early detection, and intervention-tend to yield higher long-term returns (8). These benefits can extend across the lifespan through better adult health, greater productivity, and lower future healthcare costs (10). However, current care models frequently emphasize treatment over prevention and institutional care over community-based solutions. This approach has created ongoing debates about cost-effectiveness and has limited the potential to maximize health gains while using resources efficiently (6).

Therefore, this perspective study adopts a health economics perspective to systematically compare the cost-effectiveness of nursing care for children and older adults. It examines differences in their care needs, cost structures, benefit dimensions, and time horizons. The aim is not only to advance theoretical understanding of nursing care evaluation but also to propose evidence-based strategies for optimization. The findings are intended to inform policy development, improve service design, and promote equitable, efficient allocation of health resources across generations and life stages.

Although this article is presented as a perspective piece, the conceptual arguments it advances are informed by a structured literature scan conducted in PubMed and Web of Science, covering publications from 2000 to 2025. The search strategy combined terms such as “nursing,” “cost-effectiveness,” “quality-adjusted life year,” “pediatric care,” “geriatric care,” and “long-term care financing.” Priority was given to systematic reviews, economic evaluations reporting incremental cost-effectiveness ratios (ICERs), and policy-focused analyses. The selected literature was synthesized narratively to identify recurring economic themes and methodological gaps, rather than to conduct a formal systematic review.

## 2 Cost-effectiveness analysis (CEA) framework for nursing caring for children and the older adults

**Figure 1:**
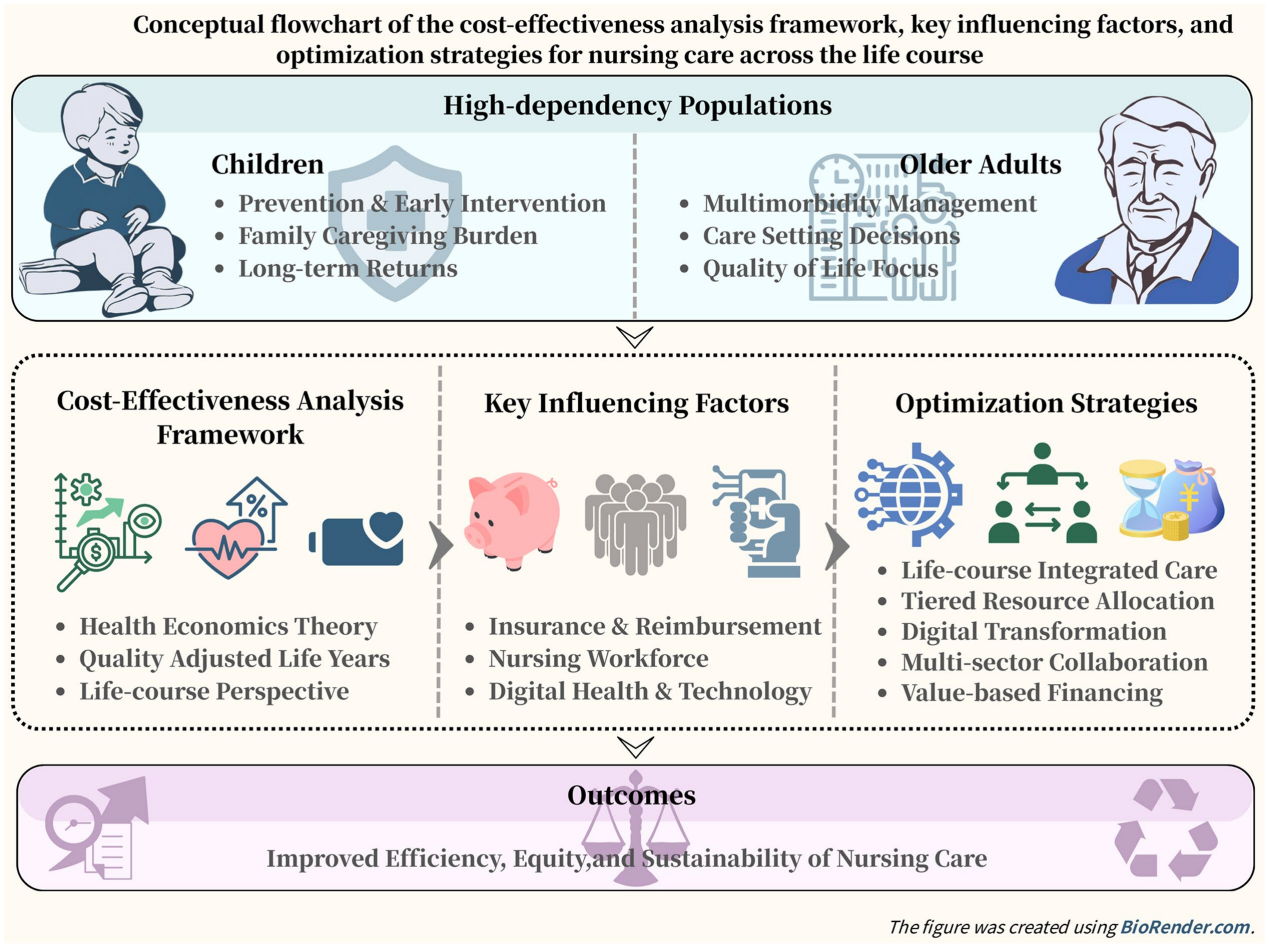
Conceptual flowchart of the cost-effectiveness analysis framework, key influencing factors, and optimization strategies for nursing care across the life course.

A CEA framework is essential for evaluating the economic efficiency of nursing care for children and the older adults. This framework systematically compares the costs of care with the resulting health outcomes, providing an evidence-based foundation for resource allocation. The following section develops a comparative analytical structure to examine these two distinct populations, establishing a theoretical basis for subsequent analysis and policy discussion (Figure 1).

### 2.1 Theoretical foundations of CEA

CEA, rooted in health economics, quantifies the resources used and the health benefits achieved by specific interventions (11). Common approaches include CEA, which measures benefits in monetary terms, and cost-utility analysis, which uses integrated measures such as quality-adjusted life years (QALYs) to combine length and quality of life (11). Applying this to nursing requires a life-cycle perspective. Health investments yield different returns across life stages: investments in children may produce benefits over decades, enhancing future productivity and reducing later healthcare costs (12), whereas investments in the older adults often focus on improving current quality of life, maintaining function, and preventing acute health events (13). Recognizing these differing return periods is central to understanding the economics of care for each group.

To enhance the analytical rigor of cost-effectiveness evaluations in nursing, it is essential to explicitly define the study perspective, time horizon, discount rate, and outcome measure (14). The perspective may be societal or that of the healthcare payer. Adopting a societal viewpoint allows for the inclusion of indirect costs, such as caregiver productivity loss, which is particularly relevant in pediatric nursing (11). The ICERs should be calculated as the difference in costs divided by the difference in health outcomes, typically measured in QALYs or other appropriate metrics (11, 14). When a long-term time horizon is applied, both costs and benefits should be discounted at a standard annual rate of 3 to 5%, a practice that can substantially influence the evaluation of early-life interventions (11, 15). Finally, a clearly defined willingness-to-pay threshold is necessary to determine whether an intervention represents acceptable value for money (16, 17).

### 2.2 Cost-effectiveness characteristics of nursing for children

Nursing for children emphasizes prevention and early intervention. Costs extend beyond direct medical expenses to include substantial indirect family costs, such as lost parental income and long-term support needs (18). The benefits, however, are long-term and broad. For example, vaccinations and nutritional support require modest investment but can prevent significant future disease and economic burden (19, 20). Early intervention for developmental or mental health concerns can improve a child’s lifelong trajectory and reduce future reliance on specialized services (21). Thus, evaluating cost-effectiveness in child nursing account for long-term socioeconomic gains and the accumulation of health capital over time.

### 2.3 Cost-effectiveness characteristics of nursing for the older adults

In contrast to child nursing, nursing for the older adults focuses on managing existing chronic conditions and providing sustained support (22). Costs are cumulative and ongoing, covering long-term medication, routine care, functional support, and end-of-life care (9, 23, 24). Benefit assessment centers on the balance between quality of life and disease burden, often captured through measures like QALYs (25). For older adults, care aims less to extend life than to enhance its quality, preserve dignity, and reduce caregiver burden (22). The key economic question becomes how to achieve the greatest improvement in quality of life within feasible cost constraints.

### 2.4 Comparative analysis

A comparative analysis of pediatric and geriatric nursing reveals significant structural and economic distinctions. In terms of input, pediatric care is frequently conceptualized as a form of prospective investment, whereas geriatric care is generally regarded as continuous management aimed at maintaining function and quality of life (12). In terms of outcomes, nursing interventions in children yield delayed, often society-wide benefits-such as increased future productivity and reduced lifetime morbidity-while those in older adults produce more immediate, individualized gains in wellbeing and functional status (8, 26). In terms of timeframe, returns on pediatric investments typically unfold over decades, requiring longitudinal evaluation frameworks, whereas the effects of geriatric interventions are usually observable within shorter time horizons, often measured in months or years (8, 26).

However, this investment-management distinction should not be interpreted as categorical or mutually exclusive. Acute pediatric care for complex congenital anomalies or rare diseases may entail extremely high upfront costs with uncertain long-term returns, thereby challenging the assumption of uniformly favorable lifetime economic yield (27, 28). Conversely, preventive strategies in older adults-such as fall prevention programs, comprehensive medication management, and community-based functional maintenanc-have demonstrated highly favorable ICERs and, in some contexts, substantial cost savings (29–31). These examples illustrate that both populations encompass preventive and high-intensity care components, each associated with distinct cost structures, risk profiles, and value trajectories.

Accordingly, rather than relying on a binary classification, we propose a temporal risk–return gradient framework. This framework conceptualizes pediatric and geriatric nursing along a continuum defined by variation in investment horizon, uncertainty of returns, and immediacy of benefit realization. Within this gradient, interventions are evaluated according to their position on the spectrum of short-term versus long-term value generation, as well as their distribution of costs and benefits across individuals and society (32, 33).

These structural and temporal distinctions have important implications for economic evaluation. They suggest that a single cost-effectiveness standard-applied uniformly across age groups-may obscure meaningful heterogeneity in value realization, discounting sensitivity, and societal return distribution (34). Effective policy should therefore be grounded in a differentiated analytical approach that accounts for life-course position, risk profile, and distributional consequences, thereby aligning resource allocation with each population’s unique economic and health characteristics (35).

## 3 Key factors influencing cost-effectiveness

Cost-effectiveness in nursing is shaped not only by the specific interventions used but also by population characteristics, care delivery models, and broader health system conditions. For children and older adults, the factors that influence outcomes differ substantially, requiring tailored approaches in both policy and practice (Figure 1).

### 3.1 Children

For children, disease patterns play a major role. Congenital conditions, developmental disorders, and rare diseases often demand early, continuous, and multidisciplinary care (36, 37). While this involves significant direct medical costs, timely investment can improve long-term health outcomes and yield substantial returns (37). Additionally, the reliance on family caregiving introduces important indirect costs, including lost parental income and interrupted careers (38, 39). These socioeconomic impacts should be factored into any evaluation of child nursing benefits. Furthermore, the accessibility and coverage of preventive services—such as immunizations and developmental screenings—are critical (40). When prevention is widely accessible, it reduces the incidence of avoidable illnesses and their long-term consequences, improving the overall efficiency of health spending.

### 3.2 Older adults

For older adults, clinical complexity and care settings strongly influence cost-effectiveness. Multimorbidity and the use of multiple medications increase the risk of treatment conflicts and adverse events, often leading to repeated tests, hospital readmissions, and higher costs (41, 42). The choice between institutional care and home- or community-based care also affects resource use. Institutional care tends to have higher fixed costs, whereas home-based care depends heavily on family and social support, which—if inadequate—can compromise quality and safety (6). Additionally, palliative and end-of-life care present distinct evaluation challenges. Their value lies less in prolonging life than in relieving symptoms, providing psychological support, and improving the quality of dying (43). This often leads to debate over how to assess benefits and allocate resources ethically in this context.

### 3.3 Systemic factors

System-level factors further shape cost-effectiveness for both groups. Health insurance systems influence out-of-pocket costs and service coverage; reimbursement policies that prioritize cost-effective prevention and early intervention can steer resources toward higher-value care (44). The nursing workforce—its distribution, skill level, and size—directly affects service quality and efficiency (45). Shortages or inadequate training may lower care standards and raise long-term risks (45). Technological advances, such as remote monitoring and assistive devices, offer potential gains in efficiency, function, and caregiver support (46, 47). However, their real-world impact depends on affordability, accessibility, and data security.

In summary, improving cost-effectiveness in nursing requires a clear understanding of the distinct factors affecting children and older adults, supported by coherent policies and well-integrated system-level conditions.

## 4 Strategies for optimizing the cost-effectiveness of nursing caring

Improving the cost-effectiveness of nursing care requires a shift away from fragmented, disease-centered models toward a coordinated, preventive, and value-based approach. Given the distinct needs of children and older adults, effective strategies should address care delivery, resource use, technology, collaboration, and financing (Figure 1).

What fundamentally distinguishes optimization strategies in pediatric and geriatric nursing is the dual dependency structure inherent in these populations (48). Unlike working-age adults, who are typically the primary beneficiaries of interventions, both children and older adults rely heavily on caregivers and public financing mechanisms (49). This dependency generates significant spillover effects that extend beyond direct patient utility, influencing family dynamics, workforce participation, and intergenerational resource allocation (50). Consequently, to accurately capture the full value of nursing interventions, cost-effectiveness evaluations in these populations should integrate broader societal factors, including caregiver burden, intergenerational transfers, and long-term social returns into their assessment frameworks (11, 51).

### 4.1 Life-course integrated care model

An integrated life-course model is foundational. Since health accumulates over a lifetime, early investments in childhood can reduce disease and disability in later years (52). Policies should therefore connect child health, chronic disease management, and older adults care, rather than treating them in isolation (53). For instance, long-term studies can link early-life interventions to health outcomes in adulthood and old age, demonstrating the extended benefits of prevention (54). Integrated health records and data sharing across sectors enable early risk detection and continuous support, lowering the overall burden of disease and cost of care across the lifespan (55, 56).

### 4.2 Precise resource allocation and tiered interventions

Resources should be allocated based on risk and need. For children, this means strengthening early screening for high-risk conditions and providing tailored plans that include medical, educational, and family support (57). For older adults, care should be tiered according to level of disability—with community-based prevention for those with mild needs and more intensive, integrated services for those with moderate to severe impairment (26). This approach directs resources where they are most effective.

### 4.3 Technological innovation and digital transformation

Technology can enhance efficiency and support. Digital tools for remote monitoring and family guidance can help manage children’s health, reduce unnecessary visits, and improve adherence (58). For older adults, smart home sensors and remote monitoring can help prevent falls, manage medications, and avoid costly emergencies (59, 60). Successful adoption depends on making these tools accessible, user-friendly, and secure, and ensuring they complement existing services (61).

### 4.4 Multi-sector collaboration and social support networks

Collaboration across sectors is essential. Children benefit when health, education, and social services work together to support their development and inclusion (62–64). For older adults, integrating medical care with social and community services—through community-based centers and home support programs—helps create a sustainable care network (65, 66). Volunteer and caregiver support programs can further extend professional capacity (67, 68).

### 4.5 Policy and financing mechanism optimization

To improve the population-level cost-effectiveness of nursing care, financing mechanisms should explicitly reward value rather than volume. In public health systems facing demographic shifts and fiscal constraints, reimbursement reform represents not merely a technical adjustment but a structural strategy to advance health equity and long-term sustainability. Transitioning from fee-for-service models toward outcomes-based payment aligns financial incentives with measurable health gains and discourages unnecessary service expansion (69). However, successful implementation requires operational clarity, transparent performance metrics, and system-wide accountability to avoid unintended consequences (70).

In pediatric nursing, outcomes-based reimbursement may include indicators such as developmental milestone attainment, vaccination coverage, and reductions in preventable hospital admissions (71). These measures reflect investments in long-term population health and the accumulation of health capital. In geriatric nursing, relevant metrics may include maintenance of functional independence, reduced fall-related hospitalizations, and prevention of adverse drug events (72). These outcomes correspond to preserving autonomy, reducing high-cost acute care utilization, and supporting continued community participation among older adults.

Despite their theoretical advantages, outcomes-based payment models face substantial implementation challenges. First, robust risk adjustment mechanisms are essential to account for baseline socioeconomic disparities, disease severity, and multimorbidity (73). Without such safeguards, providers serving disadvantaged or clinically complex populations may be penalized, thereby exacerbating existing health inequities (74). Second, attributing outcomes specifically to nursing interventions is difficult in integrated care systems where multidisciplinary teams jointly influence patient trajectories (75). Clear attribution frameworks and shared accountability models are therefore needed to ensure fairness and transparency. Third, value-based financing should guard against perverse incentives (76). There is a risk that providers may preferentially select lower-risk patients who can achieve measurable short-term improvements more easily (76). To prevent risk selection and protect vulnerable populations, blended payment models-combining capitation, bundled payments, and performance-based components-may offer greater stability while preserving incentives for quality improvement (77). Risk stratification tiers and equity-sensitive benchmarks can further ensure that high-need individuals continue to receive adequate services.

Beyond reimbursement structure, strengthening long-term care insurance systems and establishing dedicated funds for high-cost conditions-such as rare pediatric diseases or advanced frailty syndromes-are essential for financial protection and universal health coverage (78). From a public health standpoint, financial risk protection is a core system objective, particularly for dependent populations with limited earning capacity (79).

Importantly, financing reform in the pediatric-geriatric nursing context should address intergenerational equity. Pediatric interventions often generate long-term societal returns, while geriatric care yields immediate but shorter-term quality-of-life gains. Policymakers should therefore balance discounting principles, fiscal sustainability, and ethical considerations of solidarity across generations (80, 81). Incorporating age-adjusted QALYs or caregiver-adjusted utility weights-which capture spillover effects on family caregivers-may provide a more equitable evaluative framework than standardized population-level metrics alone (82).

Collectively, these financing strategies support a shift toward value-driven, equity-sensitive, and sustainability-oriented nursing systems. By aligning reimbursement with measurable health outcomes while safeguarding access for high-risk groups, health systems can enhance the cost-effectiveness of nursing care across the life course and advance public health objectives at the population level (83).

## 5 Challenges and future directions

Despite a strong rationale for improving the cost-effectiveness of nursing care for children and older adults, significant barriers remain in practice, and these point to important priorities for future work.

### 5.1 Implementation challenges

The first set of challenges is practical and methodological. Data are often fragmented, and there is no common standard for measuring costs and benefits, especially for outcomes like quality of life and family wellbeing (84). As a result, many cost-effectiveness studies rely on limited or short-term data, making it hard to assess value over a lifetime (85). Social and ethical factors also complicate implementation. Attitudes toward family responsibility, institutional care, and end-of-life support vary widely across cultures, making it difficult to apply uniform standards or policies (86). Balancing efficient resource use with respect for cultural and family autonomy remains a delicate task.

### 5.2 Research prospects

Future research should address these gaps by building a stronger, longer-term evidence base. Longitudinal studies that follow individuals over time are needed to understand how early childhood interventions or preventive care in later life affect health, social participation, and even outcomes across generations (87). Research should also become more interdisciplinary, combining insights from health economics, nursing, public health, and social sciences to better capture the full value of care—including psychosocial support and quality of life (88, 89).

### 5.3 Policy recommendations

Policymakers can respond by creating flexible, evidence-informed systems. This means setting up mechanisms to regularly assess the cost-effectiveness of nursing care and adjust resource allocation as populations and technologies change (90). It also means learning from other countries. International exchange can offer valuable models for financing long-term care, supporting community-based services, and covering high-cost conditions like rare childhood diseases (90, 91). By sharing knowledge and adapting successful approaches, health systems can move toward more sustainable, effective, and compassionate care for all ages (91, 92).

## 6 Summary

This perspective study has examined the cost-effectiveness of nursing care for children and older adults, highlighting both key differences and shared goals between the two groups. Nursing for children acts as a long-term investment, building health and social benefits over a lifetime. For older adults, care focuses more on sustaining quality of life and independence through ongoing support. While their economic and temporal profiles differ, both aim to use resources wisely to improve health and manage costs.

Effective strategies balance fairness, efficiency, and long-term sustainability. Policies should be tailored to each group’s needs while also promoting integrated, lifelong approaches to care. Payment systems should reward value and outcomes rather than volume of services. Improving the cost-effectiveness of nursing requires commitment from policymakers, health systems, professionals, families, and communities. Through coordinated effort and shared vision, we can build a more responsive, effective, and sustainable care system for all ages.

## Data Availability

The original contributions presented in the study are included in the article/supplementary material, further inquiries can be directed to the corresponding author.
